# Understanding Vaccine Hesitancy in Louisiana Through Social Media Listening and Community Feedback: Cross-Sectional Study

**DOI:** 10.2196/76827

**Published:** 2026-05-06

**Authors:** Angie Viviana Sanchez, Sadie Smith, Sahithya Sakhamuri, Julia Ardoin, Henry Chu

**Affiliations:** 1 Louisiana Center for Health Innovation (LCHI), University of Louisiana at Lafayette, 635 Cajundome Blvd, Lafayette, LA, 70506, United States, 1 337-482-0694; 2 Brigham And Women's Hospital, 75 Francis StreetBoston, MA, 02115, United States, 1 (617) 732-5500; 3 School of Computing and Informatics, University of Louisiana, Lafayette, LA, United States; 4 Informatics Research Institute (IRI), University of Louisiana, Lafayette, LA, United States

**Keywords:** vaccines, vaccine sentiments, vaccine hesitancy, social media listening, public health campaigns, Louisiana

## Abstract

**Background:**

The rise of social media has significantly impacted public health programs, with platforms such as YouTube, Facebook, X (formerly known as Twitter), Instagram, and, more recently, TikTok being used to promote health information, raise awareness about disease outbreaks, and support disease prevention programs. However, the diverse and often unverified nature of the content on social media can make it challenging to discern accurate information, contributing to user uncertainty, which may in turn contribute to low vaccination rates in some regions. This is especially true in Louisiana as its COVID-19 vaccination rates were among the lowest in the country in 2022. Therefore, understanding public sentiment on social media and developing targeted campaigns to counter unverified information is essential for advancing public health campaigns.

**Objective:**

The goal was to gain insights into the underlying factors that contribute to Louisiana’s low vaccination rates for routine immunizations by (1) performing social media listening to develop an infodemic management plan and (2) promoting accurate information via a social media campaign.

**Methods:**

Social media listening was conducted using Meltwater, a media monitoring and social media listening platform, supplemented by Google Alerts and Google News to identify if vaccine-related stories or sentiments were attracting unusual attention. Additionally, a social media campaign aimed at educating Louisiana residents about disease manifestation, symptoms, vaccines available for disease prevention, and potential side effects was developed. Posts were published 2 to 3 times a week and boosted for 7 days.

**Results:**

From November 13, 2023, to June 11, 2024, social media listening identified at least 15 unique, noteworthy stories that signified sentiment spikes. These conversations were predominantly related to vaccine hesitancy, with users expressing opposition to vaccines or reluctance to engaging with vaccine-related information. Sentiment spikes included themes related to mistrust of vaccines and concerns about their safety and efficacy. The social media campaign received 69,600 impressions, reached 43,429 users, and received 652 reactions and likes, 62 shares, and 105 comments. Most of the audience was female, with higher engagement from older users on Facebook and younger users on Instagram. Finally, posts related to hepatitis B, rotavirus, and measles, mumps, and rubella vaccines received the most attention.

**Conclusions:**

Social media has become a key tool for digital health, helping to implement disease prevention programs and promoting advances in medicine. However, unverified information remains a major reason for the aversion to vaccination despite the dissemination of information from reputable public health organizations, health professionals, hospitals, and medical centers. To address this, information that is accessible, understandable, and culturally competent must be circulated to mitigate disinformation and improve attitudes toward vaccination. More research is needed to evaluate the effectiveness of social media campaigns in reducing vaccine hesitancy and improving willingness to adopt public health recommendations to increase vaccination rates.

## Introduction

Since its popularization in the early 2000s, social media has evolved into a multifaceted tool for multiple uses: socializing with friends, family, and the public; professional networking; and accessing information in conjunction with traditional news media. Presently, social media has penetrated all generations as it offers a variety of options, including engaging with videos, entertainment, news, opinion forums, and more. Platforms such as YouTube, Facebook, X (formerly known as Twitter), Instagram, and, recently, TikTok have been used not only as spaces for connection and community but also as a means of disseminating information [1-3].

The emergence of new social media platforms and applications has substantially impacted public health strategies. Health care providers, public health professionals, and other organizations began using social media for health education campaigns to issue timely information about disease outbreaks and inform target audiences about varying health topics [4,5]. Furthermore, with advancements in medical technology, these platforms have become an increasingly fundamental part of medical research. Researchers now use social media to gain insights into public attitudes, such as trust in medical personnel, acceptance of new treatments or prevention mechanisms (eg, vaccines), and hesitancy toward adopting public health recommendations [5,6].

While skepticism and doubt toward vaccinations have been present and influential throughout history [7-9], the emergence of social media has amplified these challenges [3]. As an easily accessible, user-driven information channel, social media enables a rapid flow of content that can be overwhelming, confusing, and anxiety inducing [2,6]. The abundance of accurate, false, and contradictory information being shared creates a breeding ground for misinformation [1,3,10]. For example, during the COVID-19 pandemic, the rapid spread of unverified information on social media generated high levels of confusion, anxiety, and mainstream rejection of vaccines and prevention guidelines disseminated by health personnel and public health organizations [11-13]. Although distrust is not a new barrier in public health, the rapid spread of inaccurate information on social media has become a contributing factor to low vaccination rates in some regions [14-17]. For instance, in 2022, only 56% of Louisiana’s population was vaccinated against COVID-19, ranking it as the state with the fourth lowest vaccination rate in the United States [18,19]. This gap is especially evident when comparing Louisiana to other states in the United States. In 2023, states such as Rhode Island, Massachusetts, the District of Columbia, and Hawaii each reported that 80% or more of their population were fully vaccinated against COVID-19, with over 38% having received a booster dose. In contrast, less than 1% of Louisiana’s population had received a booster dose the same year [19].

Vaccination gaps extend past COVID-19 and are present in routine childhood and influenza immunizations. These disparities often vary by state. For example, during the 2022 to 2023 influenza season, only 41.1% of Louisiana’s residents were vaccinated, falling below the national average of 46.9%, whereas states such as Massachusetts reached 62.4% [20]. Similarly, uptake of the measles, mumps, and rubella (MMR) vaccine, which is part of routine childhood vaccinations, declined by almost 30% among children with Medicaid in Louisiana in 2020 compared to previous years, perhaps as a consequence of the COVID-19 pandemic [21]. Additionally, racial gaps in childhood vaccination rates persisted at the state level from 2019 to 2021. In Louisiana, African American children were almost 12% less likely to have received the full schedule of recommended vaccinations compared to White children [22]. Additionally, 76.1% of privately insured children aged 35 months received all the recommended vaccines in 2020 to 2021 compared to 57.8% of children with Medicaid. In 2021, Louisiana ranked fifth lowest in childhood vaccination coverage with a rate of 62% compared to states such as Iowa, Massachusetts, Connecticut, Vermont, and North Dakota, all of which reported vaccination rates exceeding 80% [22].

With guidance from the World Health Organization’s (WHO) Infodemic Management training and the social media listening and monitoring tools from the US Department of Health and Human Services Centers for Disease Control and Prevention (CDC), this study aimed to gain insights into the factors that contribute to Louisiana’s low vaccination rates [23,24]. Using these resources, a social media listening plan was developed to analyze circulating conversations about vaccines. Using the insights from social media listening, a social media educational campaign was developed. We hypothesized that this campaign could help reduce erroneous information regarding vaccines and promote vaccine confidence. This approach aimed to empower individuals to make informed decisions without fear or false claims of adverse events. This paper describes the search strategies used to perform social media listening, topics covered in the health and vaccination educational campaign, and the resulting levels of user engagement and acceptance.

## Methods

### Overview

This was a qualitative, cross-sectional study assessing vaccine hesitancy through social media listening and a social media campaign. First, social media listening took place from November 13, 2023, to June 11, 2024. After 5 months, the insights gathered were used to develop a social media educational campaign that was implemented from April 22, 2024, to June 28, 2024.

### Ethical Considerations

This study did not undergo institutional review board approval as it used publicly available social media data for the analysis, which were obtained from Facebook, Instagram, X, and Meltwater under their terms of service. Names or other identifiable information were not collected. Instead, the study used age and gender cluster data provided by Meta Analytics for Facebook and Instagram.

### Social Media Listening

To understand the online conversation regarding vaccines, Meltwater—a media monitoring and social listening platform—was used to perform social media listening, occasionally supplementing search results using Google Alerts and Google News depending on the capabilities of social media listening tools. In total, 12 saved searches grouped by topic and 1 saved search limited to Louisiana-based social media users were created. Saved searches were built to accurately capture vaccine sentiment. Each search was constructed using a series of keywords separated by Boolean operators to narrow down the focus of the results. For example, to explore discussions about vaccines related to opinions on the credibility of the CDC and the Food and Drug Administration, the following Meltwater search was built: “(‘vax’ OR ‘vaccine*’) AND (‘Center for Disease Control’ OR ‘CDC’ OR ‘FDA’ OR ‘Food and Drug Administration’) AND (‘lie*’ OR ‘credibility’ OR ‘trust’ OR ‘conspiracy’ OR ‘conspiracies’ OR ‘approval process’ OR ‘approval transparency’ OR ‘approval credibility’ OR ‘Fauci’ OR ‘World Health Organization’ OR ‘National Institute of Health’).” The search was limited to return results that were in English and from US-based sources. These searches were developed and grouped by the following topics: CDC and Food and Drug Administration credibility, childhood vaccines, COVID-19 in the United States, influenza vaccine in the United States, Louisiana Department of Health, public health agencies (eg, CDC and WHO), respiratory syncytial virus (RSV) vaccine, rural health care, vaccine alternatives, vaccine brands, and vaccine hesitancy: general United States and vaccine hesitancy: Louisiana. All Meltwater saved searches and keywords can be found in Multimedia Appendix 1.

Initial queries were built iteratively with guidance from Meltwater advisors, who provided the best practices for optimizing social media listening searches on their platform. Team members then refined these queries to better align with the study’s goal of capturing themes and opinions that offered insights into the general public’s feelings and attitudes toward vaccines. Team members attended meetings with Meltwater to review search terms and occasionally edited them to improve the sensitivity and specificity of the searches. Team members sifted through search results daily using Meltwater’s tagging feature to save noteworthy results and met biweekly to review saved posts and discuss discrepancies. Sentiment classification was conducted using Meltwater’s automated sentiment analysis feature, which categorizes content as positive, neutral, or negative based on algorithms and manually. Team members would first allow Meltwater to apply its automated sentiment analysis and would then reclassify sentiments based on perceived tone, language, and overall message content. Every 2 weeks, a team member built a dashboard in Meltwater using analytics tools that combined the various search results into consumable insights, including top keywords, total mentions, total mentions trend, and sentiment breakdown. These dashboards highlighted instances when vaccine-related stories or sentiments were receiving higher-than-average levels of online engagement.

### Social Media Campaign

From social media listening, one of the biggest issues identified that contributed to vaccine hesitancy was a general sense of misunderstanding vaccines and their role in preventing disease. To remedy this information gap, a vaccine education campaign was created. To conduct the campaign, social media accounts were created on Facebook, Instagram, and X under the name @ECHOatLCHI (“ECHO” stands for Empowering Communities for Healthy Outcomes and “LCHI” stands for Louisiana Center for Health Innovation), with the goal of providing general education about vaccines to Louisiana residents. To keep the audience engaged without overloading social media accounts, informative posts were published on these accounts 2 to 3 times a week and boosted for 7 days on Facebook and Instagram. Approximately US $15 were spent per post on each account. Social media interactions and impressions were tracked over the duration of the social media campaign. One member of the research team systematically reviewed all user comments on Facebook and Instagram posts and classified them into 2 sentiment categories (positive and negative) based on content and tone.

The included posts addressed fundamental questions such as “What is a vaccine?” “How do vaccines work?” and “Why are vaccines necessary?” (Figure S1 in Multimedia Appendix 2). From this, the content shifted to providing education about target vaccines: the influenza vaccine (Figure S2 in Multimedia Appendix 2), routine childhood vaccines (Figure S3 in Multimedia Appendix 2), and the RSV vaccine (Figure S4 in Multimedia Appendix 2). To align with current campaigns led by the Louisiana Department of Health and regional medical directors, information related to human papillomavirus (HPV) was included at the end of this campaign. Each post covered information related to disease manifestation, symptoms, available vaccines for prevention, and vaccine side effects. The target audience for the boosted posts included users in Louisiana aged 18 years and older. From this campaign, we intended to record the number of impressions we received for each post and also the basic demographic information of the target audience. Demographics such as gender and age range were captured using Meta Analytics for Facebook and Instagram. Unfortunately, X did not provide this information, limiting the analysis to Facebook and Instagram.

## Results

### Social Media Listening

Every 2 weeks, 2 to 3 noteworthy news stories or sentiments that attracted increased social media attention were highlighted. During the course of social media listening, up to 15 unique, noteworthy stories that signified a sentiment spike were identified (Table 1). On the basis of the analysis, most social media conversations about vaccines involved users who were either opposed to vaccines or uninterested in engaging with vaccine-related content. Generally, vaccine conversations often revolved around certain major, underlying themes, including mistrust of the government; pharmaceutical companies; the CDC, the WHO, and other public health organizations; mistrust of vaccine safety and efficacy; and health freedom (belief in bodily autonomy over mandatory vaccination).

**Table 1.** Stories or sentiments that were attracting increased social media attention.

Sentiment or story	Reporting time frame
RSV^a^ vaccine awareness and lack of awareness	November 13 to 27, 2023
Texas Attorney General Ken Paxton announced plans to sue Pfizer	November 28, 2024, to December 11, 2024
A public health worker in New Zealand “proved” excess death caused by COVID-19 vaccines	November 28, 2024, to December 11, 2024
Rise in respiratory illness (COVID-19 and influenza) cases	December 25, 2023, to January 8, 2024
Florida Surgeon General Dr Joseph Ladapo’s comments against mRNA^b^ COVID-19 vaccine	January 9 to 22, 2024
Clusters of measles outbreaks generated conversations about childhood vaccines	January 9 to 22, 2024; February 20, 2024, to March 4, 2024; March 5 to 18, 2024; and March 19, 2024, to April 1, 2024
Mistrust toward the CDC^c^, the WHO^d^, and pharmaceutical companies^e^	January 23, 2024, to February 5, 2024; April 16 to 29, 2024; and May 13 to 27, 2024
Reports of COVID-19 vaccine side effects and injury^e^	February 6 to 19, 2024; March 5 to 18, 2024; April 2 to 15, 2024; and May 14 to 27, 2024
New COVID-19 isolation and vaccine guidance from the CDC	February 20, 2024, to March 4, 2024
Inaccurate information related to blood donation and COVID-19 vaccination status	February 20, 2024, to March 4, 2024
Louisiana legislature surrounding vaccines and Louisiana school vaccine opt-out rates	March 19, 2024, to April 1, 2024
H5N1 avian influenza	April 2 to 15, 2024; April 16 to 29, 2024; April 30, 2024, to May 13, 2024; and May 14 to 27, 2024
AstraZeneca withdrew its COVID-19 vaccine from the market	April 30, 2024, to May 13, 2024
Dr Anthony Fauci testified in Congress in COVID-19–related hearing	May 28, 2024, to June 10, 2024
Moderna developing combination influenza and COVID-19 vaccine	May 28, 2024, to June 10, 2024

a RSV: respiratory syncytial virus.

b mRNA: messenger RNA.

c CDC: Centers for Disease Control and Prevention.

d WHO: World Health Organization.

e Consistent, overarching theme in vaccine hesitancy conversations.

In addition to the sentiment spikes, we highlighted “information gaps” (Table 2), which were areas where social media users lacked accessible, credible information from trustworthy sources. In the absence of credible information, users often create and adopt unsubstantiated claims as an answer. Information gaps can be problematic as they can grow rapidly and fuel misunderstanding if not properly addressed by credible sources.

**Table 2.** Information gaps on social media among users without accessible, credible information from legitimate sources.

Information gap	Reporting time frame
RSV^a^ vaccine: eligibility, availability, and how it works	November 13 to 27, 2023, and December 25, 2023, to January 8, 2024
Novavax: differences from other COVID-19 vaccines, effectiveness, and availability	November 28, 2023, to December 11, 2023
Vaccine cost and insurance coverage	November 28, 2023, to December 11, 2023
Vaccine “shedding” and other general questions about vaccine technology	January 9 to 22, 2024, and April 30, 2024, to May 13, 2024
mRNA^b^ technology and safety	January 23, 2024, to February 5, 2024; February 6 to 19, 2024; and May 28, 2024, to June 10, 2024
MMR^c^ vaccination and necessity	February 20, 2024, to March 4, 2024; March 5 to 18, 2024; and March 19, 2024, to April 1, 2024
New CDC^d^ guidance on COVID-19	February 20, 2024, to March 4, 2024
H5N1: should I be concerned? How can I prevent it?	April 2 to 15, 2024; April 16 to 29, 2024; April 30, 2024, to May 13, 2024; and May 14 to 27, 2024
General necessity of vaccines vs just trusting one’s immune system	May 28, 2024, to June 10, 2024

a RSV: respiratory syncytial virus.

b mRNA: messenger RNA.

c MMR: measles, mumps, and rubella.

d CDC: Centers for Disease Control and Prevention.

### Social Media Campaign

Overall, the social media posts received 69,600 total impressions (calculated by the number of views that all posts received across all platforms; Table 3); reached 43,429 users (the number of users who saw any post); and received 652 reactions and likes, 62 shares, and 105 comments. Posts related to general vaccine information on Facebook reached a higher proportion of older users (aged 55 years and older), whereas posts on Instagram reached younger audiences (Table 3). Reach metrics were limited to Facebook and Instagram as X does not provide this metric for organic posts.

**Table 3.** Total impressions and reach on social media across different platforms, along with the most predominant age group reached, the corresponding number of users reached for each platform, and reactions.

Post ID and social media platform	Users reached, n	Impressions, n	Most prominent age range reached (y)	Users reached in this age range, n/N (%)	Comments, n	Shares, n	Total reactions, n	Breakdown of reactions
General 1								
Facebook	1609	2601	≥55	545/1609 (33.87)	0	2	3	3 likes
Instagram	828	1978	25-34	348/828 (42.03)	—^a^	—	—	—
X	—	19	—	—	—	—	—	—
General 2								
Facebook	455	821	≥55	227/455 (49.89)	12 (11 negative or spam)	5	12	5 likes, 3 laughs, 3 angry reactions, and 1 sad reaction
Instagram	925	1856	18-24	511/925 (55.24)	—	—	—	—
X	—	21	—	—	—	—	—	—
General 3								
Facebook	672	1192	18-24	370/672 (55.06)	0	2	2	2 likes
Instagram	695	1077	18-24	337/695 (48.49)	—	—	—	—
X	—	21	—	—	—	—	—	—
General 4								
Facebook	1496	2349	≥55	595/1496 (39.77)	0	3	4	3 likes and 1 angry reaction
Instagram	826	1976	25-34	330/826 (39.95)	—	—	—	—
X	—	23	—	—	—	—	—	—
General 5								
Facebook	789	1106	≥55	582/789 (73.76)	16 (all negative or spam)	8	62	47 likes, 10 laughs, 2 loves, 2 angry reaction, and 1 care reaction
Instagram	805	1307	18-24	266/805 (33.04)	—	—	—	—
X	—	31	—	—	—	—	—	—
General 6								
Facebook	633	864	≥55	243/633 (38.39)	1 (all negative or spam)	1	48	45 likes, 2 loves, and 1 laugh
Instagram	827	1752	25-34	353/827 (42.68)	—	—	—	—
X	—	18	—	—	—	—	—	—
General 7								
Facebook	626	912	≥55	425/626 (67.89)	8 (all negative or spam)	1	58	33 likes, 17 laughs, 6 angry reactions, 1 care reaction, and 1 sad reaction
Instagram	1006	1882	18-24	533/1006 (52.98)	—	—	—	—
X	—	15	—	—	—	—	—	—
General 8								
Facebook	589	806	≥55	251/589 (42.61)	10 (all negative or spam)	0	56	45 likes, 9 laughs, and 2 angry reactions
Instagram	1085	1945	18-24	445/1085 (41.01)	—	—	—	—
X	—	16	—	—	—	—	—	—
General 9								
Facebook	397	442	≥55	240/397 (60.45)	0	0	53	49 likes, 3 laughs, and 1 care reaction
Instagram	1141	1858	18-24	485/1141 (42.51)	—	—	—	—
X	—	12	—	—	—	—	—	—
General 10								
Facebook	321	391	≥55	189/321 (58.88)	6 (all negative or spam)	0	56	54 likes, 1 love, and 1 angry reaction
Instagram	1247	2101	18-24	555/1247 (44.51)	—	—	—	—
X	—	18	—	—	—	—	—	—
Influenza 1								
Facebook	2076	2849	25-34	958/2076 (46.15)	1 (all negative or spam)	7	14	11 likes, 2 loves, and 1 “wow” reaction
Instagram	711	1465	25-34	259/711 (36.43)	—	—	—	—
X	—	17	—	—	—	—	—	—
Flu 2								
Facebook	468	657	≥55	151/468 (32.26)	3 (2 negative or spam)	2	58	54 likes, 3 loves, and 1 care reaction
Instagram	723	1254	18-24	235/723 (32.50)	—	—	—	—
X	—	16	—	—	—	—	—	—
Childhood 1								
Facebook	3262	4589	25-34	2158/3262 (66.16)	3 (all negative or spam)	4	2	2 likes
Instagram	867	1436	25-34	289/867 (33.33)	—	—	—	—
X	—	32	—	—	—	—	—	—
Childhood 2								
Facebook	1561	1944	≥55	935/1561 (59.90)	1 (negative or spam)	0	44	41 likes, 2 loves, and 1 care reaction
Instagram	998	1337	25-34	352/998 (35.27)	—	—	—	—
X	—	22	—	—	—	—	—	—
Childhood 3								
Facebook	1983	2737	25-34	1147/1983 (57.84)	5 (all negative or spam)	5	7	7 likes
Instagram	796	1674	25-34	336/796 (42.21)	—	—	—	—
X	—	23	—	—	—	—	—	—
Childhood 4								
Facebook	1120	1551	≥55	743/1120 (66.34)	20 (19 negative or spam)	0	70	65 likes, 3 loves, 1 laugh, and 1 angry reaction
Instagram	955	1597	18-24	399/955 (41.78)	—	—	—	—
X	—	21	—	—	—	—	—	—
Childhood 5								
Facebook	1376	1886	25-34	721/1376 (52.40)	4 (3 negative or spam)	6	14	12 likes and 2 loves
Instagram	869	1541	25-34	332/869 (38.20)	—	—	—	—
X	—	29	—	—	—	—	—	—
Childhood 6								
Facebook	2250	2894	≥55	684/2250 (30.40)	11 (all negative or spam)	13	37	23 likes, 7 angry reactions, 4 laughs, 2 sad reactions, and 1 love
Instagram	1001	1545	25-34	353/1001 (35.26)	—	—	—	—
X	—	23	—	—	—	—	—	—
RSV^b^ 1								
Facebook	1193	1814	25-34	586/1193 (49.12)	0	2	43	39 likes, 3 loves, and 1 care reaction
Instagram	882	1759	25-34	359/882 (40.70)	—	—	—	—
X	—	21	—	—	—	—	—	—
RSV 2								
Facebook	296	361	18-24	112/296 (37.84)	1 (all negative or spam)	0	3	3 likes
Instagram	748	1657	25-34	338/748 (45.19)	—	—	—	—
X	—	13	—	—	—	—	—	—
HPV^c^ 1								
Facebook	1784	2530	25-34	690/1784 (38.68)	3 (all negative or spam)	1	6	6 likes
Instagram	538	873	25-34	185/538 (34.39)	—	—	—	—
X	—	23	—	—	—	—	—	—
Total	43,429	69,600	—	—	105	62	652	549 likes, 48 laughs, 23 angry reactions, 21 loves, 6 care reactions, 4 sad reactions, and 1 “wow” reaction

a Not available.

b RSV: respiratory syncytial virus.

c HPV: human papillomavirus.

The most prominent and highly reached audience segment consisted of women aged 25 to 34 years, representing 28.14% (12,221/43,429) of the total reached audience (Table 4). Across Facebook and Instagram, posts achieved a higher reach and received a higher number of impressions and greater engagement among women than among men (Table 5). Facebook posts received significantly higher engagement than the other platforms. Posts focused on routine childhood vaccines outperformed posts related to general vaccine information and posts about influenza, RSV, and HPV in terms of engagement. The post related to hepatitis B and rotavirus vaccines (Childhood 1) achieved the highest reach and received the highest number of impressions, reaching 13.95% (6057/43,429) of the users and receiving 5.93% (4129/69,600) of the impressions across all platforms (Facebook, Instagram, and X; Table 3). The post related to the MMR vaccine (Childhood 4) garnered the most reactions and likes, receiving 20 comments, 65 likes, 3 love reactions, 1 “laugh” reaction, and 1 “angry” reaction across all platforms. Finally, the post with the most shares was about the childhood vaccine schedule (Childhood 6), receiving 13 shares across all platforms (Table 3).

**Table 4.** Demographic composition of the users reached on social media Empowering Communities for Healthy Outcomes accounts (n=43,429).

Age range (y) and gender	Users reached, n (%)
18-24	
Female	6168 (14.20)
Male	3234 (7.45)
Unknown	112 (0.26)
25-34	
Female	12,221 (28.14)
Male	2682 (6.18)
Unknown	76 (0.17)
35-44	
Female	6147 (14.15)
Male	1209 (2.78)
Unknown	53 (0.12)
45-54	
Female	2914 (6.71)
Male	718 (1.65)
Unknown	17 (0.04)
55-64	
Female	3007 (6.92)
Male	778 (1.79)
Unknown	13 (0.03)
≥65	
Female	3015 (6.94)
Male	1035 (2.38)
Unknown	30 (0.07)

**Table 5.** Total reach and impressions across Facebook and Instagram by gender based on the total reach and impressions across both platforms.

Gender	Users reached (n=43,429), n (%)	Impressions (Facebook, Instagram, and X) (n=69,166), n (%)
Female	33,472 (77.07)	51,550 (74.53)
Male	9656 (22.23)	17,187 (24.85)
Unknown	301 (0.69)	429 (0.62)

Of 105 comments, 84 (80%) expressed mistrust, negative attitudes, and hesitancy toward vaccines, and 17 (16.19%) were spam (Table 3). Examples included comments such as “Flu prevention comes from vitamins, juices, and sunshine,” “Complications including death come from vaccinations,” and “Many of us were skeptical about vaccinations before COVID-19, but now we know we can no longer trust any vax or any organization or doctor that pushes them.” To minimize the risk of influencing other users, all negative comments were hidden.

## Discussion

### Principal Findings

With the advancement of communication technologies, the medicine and public health fields have recognized the need to adopt and evolve the ways in which health messages are delivered to communities. Digital health has become vital in communicating health threats to the public; educating them about disease prevention; and promoting advances in medicine, such as new treatments [10]. Despite their potential for disseminating valuable information, social media networks have also widened gaps in access to health. This is due to an increase in skepticism, distrust, and inaccurate information caused by the overwhelming flow of true, false, and contradictory information, exacerbating historical distrust in the government and health care providers.

During social media listening in this study, most social media conversations related to vaccines included narratives related to vaccine opposition or lack of interest in engaging with vaccine-related content. Additionally, mistrust of the government, pharmaceutical companies, the CDC, the WHO, and other public health organizations; mistrust of vaccine safety and efficacy; and health freedom (belief in bodily autonomy over mandatory vaccination) were common themes identified. This indicates that there is tremendous vaccine hesitancy among social media users, which can be dangerous and counterproductive in improving vaccine uptake, especially in vulnerable populations. Regarding information gaps, the findings indicated that social media users created and adopted unsubstantiated claims. If not swiftly addressed by credible sources, information gaps can expand and drive widespread misunderstanding. These findings can be used to inform the development of tailored vaccine messaging in Louisiana.

The demographic differences in platform engagement in this study have important implications for designing targeted public health interventions. Specifically, Facebook attracted an older demographic, whereas Instagram users tended to be between the ages of 18 and 34 years. In the breakdown of demographics of users engaged with the different vaccines posts, individuals were mostly between the ages of 25 and 34 years, with Facebook attracting a slightly older demographic for the childhood vaccination posts. This aligns with existing literature on age-related patterns in social media use and suggests that public health campaigns aiming to address vaccine hesitancy should tailor their messaging strategies accordingly [25-27]. For instance, interventions targeting older adults may benefit from prioritizing Facebook as the primary source of dissemination, whereas campaigns focused on younger populations may achieve more engagement and impact using Instagram. Understanding these platform-specific audience profiles is necessary for optimizing the delivery and effectiveness of health communication efforts, especially in the context of evolving digital media landscapes.

Furthermore, the findings highlight that women aged 25 to 34 years represented the most prominent and widely reached group across the total reached audience. Similarly, posts across Facebook and Instagram consistently achieved higher reach, a higher number of impressions, and greater engagement among women compared to their male counterparts. This is also supported by the literature, which indicates that women are more likely to use social media compared to men and more likely to express their opinions on various topics through these platforms [28-30]. Among all social media platforms, posts on Facebook received the highest engagement. Posts focusing on routine childhood vaccines demonstrated higher engagement than those addressing general vaccine information, influenza, RSV, and HPV. Among these, the post concerning the hepatitis B and rotavirus vaccines achieved the highest reach and number of impressions. The MMR vaccine post received the greatest number of reactions and likes, whereas the post detailing the childhood vaccine schedule was shared the most. Overall, most of the comments received reflected mistrust, negative sentiments, and vaccine hesitancy. Scientific literature indicates that analyses of social media content frequently reveal predominantly negative sentiment regarding vaccines [31-35].

Unlike other platforms, X has been widely used in research studies due to its relatively open access to audience and content data. In contrast, popular platforms such as YouTube, Facebook, Instagram, and TikTok have stricter privacy policies. These policies enforce more stringent data sharing regulations, which increase limitations to analyzing opinions and generating universally effective strategies [10,17,36,37]. While CrowdTangle—an insight tool that gathers and analyzes social media content [38,39]—was previously used to import user activity data from Facebook, Meta began limiting its access and eventually ended all data collection activities in mid-2024 [40]. This has led to even greater limitations in gathering information from social media and understanding people’s perspectives on health prevention programs and treatments.

Despite these limitations, the research findings outlined that online vaccine sentiments are dependent on several factors, including what social media platform is more frequently used (different environments of Facebook vs Instagram vs X vs online forums), if the user stays within their algorithmically created echo chamber (users are more likely to post about a topic if their peers are also engaged), and whether vaccines are currently part of mainstream news coverage (users may not actively think about vaccines or feel encouraged to post about them until they are prompted to do so by current news stories). Past medical abuses and inconsistencies in the delivery of information have caused mistrust in many communities, especially those in underserved conditions [41-43]. Therefore, in addition to focusing on emergency response, education, and disease prevention, public health campaigns need to rebuild trust within the population. It is essential to recognize that different populations require tailored approaches depending on demographic characteristics, language, culture and religious beliefs, and needs [44-48]. Ensuring that information is accessible and understandable by everyone is crucial in public health campaigns that focus on reducing vaccine hesitancy.

### Future Directions

To maximize the reach of messages, campaigns should target a variety of media sources and social media platforms, which may engage a more diverse population. According to these findings, Facebook should be prioritized. Additionally, collaborating with interdisciplinary teams such as marketing professionals, biostatisticians, educators, and researchers can further enhance the effectiveness of campaigns. Furthermore, there is a need for more research studies to evaluate the impact of social media campaigns in reducing hesitancy and increasing the adoption of public health recommendations. This will lead to the development of more community-based research approaches, which may offer additional perspectives on strategies that improve health and digital literacy by using a more cultural and linguistic approach, helping reduce barriers to trust and improve the credibility and performance of public health campaigns on social media. These studies should incorporate a more robust evaluation design, such as a pretest-posttest assessment or the use of a comparison group, which would strengthen the ability to assess the campaign’s impact.

### Limitations

There are several limitations to the social media listening research conducted on the Meltwater platform. The absence of a baseline comparison meant that it was challenging to determine shifts in volume or sentiment over time. Future studies should incorporate a comprehensive evaluation framework as it would enable a more complete understanding of how educational content may contribute to shifts in public attitudes and understanding regarding vaccination. In addition, Meltwater could not obtain access to private social media groups such as those on Facebook, which could have omitted valuable discussion. Strict data collection restrictions on major platforms such as Facebook, Instagram, and YouTube limited the dataset mostly to X, news sites, blogs, and open forums. This constraint may introduce sampling bias and reduce the generalizability of the findings. Expanding data sources to include additional social media platforms could provide a more comprehensive understanding of vaccine hesitancy in the region and better align interventions with the audiences that need them the most. Finally, the short follow-up period after the start of social media listening may not have registered longer-term trends or shifting public sentiment as the attitudes toward vaccination can change with respect to emerging information and changing guidelines. These limitations point to the need to supplement social media listening with other research methods to have a complete view of knowledge and behavioral intentions related to vaccination.

The social media campaign and its analysis faced limitations as well. Relying on metrics of engagement such as likes, shares, comments, and impressions does not necessarily equate to the acquisition of knowledge and change in attitude or behavior regarding vaccination. Furthermore, the absence of a baseline or control group makes it difficult to attribute observed sentiment or engagement change to the campaign. Future studies should assess impact by incorporating a pretest-posttest assessment or a comparison group. Moreover, the brief 9-week campaign duration may not have been enough time to create permanent shifts in the attitudes or actions of people, so it is vital to implement a longer evaluation period to determine the true effect. Furthermore, there is a potential bias introduced by hiding negative comments, which may have skewed sentiment toward more positive or neutral responses. Future studies should aim to include all relevant user comments, including negative ones, to provide a more balanced and accurate assessment of public sentiment and engagement. Finally, the manual classification of social media comments into positive and negative sentiment by a single team member introduces the potential for researcher bias. Future studies should consider using multiple independent coders and calculating intercoder reliability or using a validated sentiment analysis tool.

## Supplementary material

10.2196/76827 Multimedia Appendix 1 Meltwater: saved searches and keywords.

10.2196/76827 Multimedia Appendix 2 Social media campaign examples.

## Abbreviations

CDC: Centers for Disease Control and Prevention
HPV: human papillomavirus
MMR: measles, mumps, and rubella
RSV: respiratory syncytial virus
WHO: World Health Organization
