# Supplementary material for: Understanding Vaccine Hesitancy in Louisiana Through Social Media Listening and Community Feedback: Cross-Sectional Study
Source: JMIR Infodemiology. 2026 May 6;6:e76827. doi: 10.2196/76827 (PMC13148589; doi:10.2196/76827)
Supplement: Multimedia Appendix 1 [file infodemiology-v6-e76827-s001.docx]

**Meltwater: Saved Searches and Keywords**

1. Public Health Agencies (CDC + WHO, etc.): ("World Health Organization" OR "CDC" OR "Centers for Disease Control" OR "National Institutes of Health" OR "Department of Health and Human Services" OR "HHS" OR "Louisiana Department of Health" OR "LDH") AND ("vaccine safety" OR "vaccine side effects" OR "vaccine risks" OR "vaccine benefits" OR "vaccine efficacy" OR "vaccine effectiveness" OR "vaccine mandates" OR "vaccine hesitancy" OR "vaccine refusal" OR "vaccine confidence" OR "vaccine trust" OR "vaccine safety concerns" OR “vaccine rights” OR “no to vaccines” OR “vaccine mandate” OR "anti-vaxxer” OR “vax” OR “vaxxed” OR “vaccine” OR "vaccine refusal" OR "vaccine misinformation" OR "vaccine skepticism" OR “covid hoax” OR “mask mandate” OR “COVID propaganda” OR “no forced vaccines” OR “unvaccinated” OR “no vax”)
2. CDC & FDA Credibility: ("vax" OR "vaccine*") AND ("Center for Disease Control" OR “CDC" OR “FDA" OR "Food and Drug Administration") AND ("lie*” OR “credibility” OR “trust” OR "conspiracy" OR "conspiracies" OR "approval process” OR “approval transparency” OR “approval credibility" OR “Fauci” OR “World Health Organization” OR "National Institute of Health")
3. Childhood Vaccines: ("childhood vaccine" OR "childhood vax" OR "child" OR "kid*" OR "autism") AND ("measles" OR "mumps" OR "MMR" OR "chickenpox" OR "diphtheria" OR "hib" OR "hepatitis A" OR "hepatitis B" OR "pertussis" OR "whooping cough" OR "polio" OR "pneumococcal" OR "rotavirus" OR "rubella" OR "tetanus" OR "HPV")
4. COVID-19 in U.S.: ("COVID vaccine" OR "COVID19") AND ("propaganda" OR "hoax" OR “religious exemption” OR “no forced vaccines” OR “unvaccinated” OR “no vax” OR “vaccine mandate” OR "long term effects of vaccine” OR “long term effects of COVID” OR "Long COVID" OR "mask mandate" OR "whistleblower*" OR "health freedom" OR "free thinker")
5. Flu Vaccine in U.S.: (“Flu vaccine” OR “flu shot” OR "flu vax" OR "influenza") AND (“mRNA vaccine" OR "infectious disease” OR “religious exemption” OR “no forced vaccines” OR “unvaccinated” OR “no vax” OR “vaccine mandate” OR "long term effects of vaccine” OR “side effects” OR "adverse effects")
6. LDH: ("Louisiana Department of Health") OR ("LDH" near/20 (health or healthcare or vaccin* or covid or flu or patient* or disease or infect* or virus*)) not ("ldh" and "schisto*")
7. RSV Vaccine: "RSV" AND "vaccine*"
8. Rural Healthcare: (“Rural health” OR “health disparities” OR “racial disparities” OR “religious conflict” OR “ethnic disparities” OR “political disparities” OR “health equity” OR “child poverty” OR “health quality of life” OR “access to the internet” OR “access to information” OR “Big pharma” OR “pharma* companies” OR “experimental vaccines” OR "trust in pharma* research" OR "trust in pharma* companies" OR "belief in pharma* research" OR "confidence in pharma* companies" OR "satisfaction with pharma* research" OR "public opinion on pharma* research" OR “health outcomes” OR “epidemic” OR “vaccine FDA approval”) NEAR/15 ("healthcare")
9. Vaccine Alternatives: ("Natural immunity" OR "Herd immunity" OR "Immune system support" OR "Homeopathic prophylaxis" OR "Vitamin supplement*" OR "Probiotics" OR "Ayurvedic medicine" OR "Traditional remed*" OR "Herbal remed*" OR "Holistic health" OR "Nutrition supplement*" OR "Naturopathic alternative*" OR "Acupuncture" OR "Homeopathy" OR "Traditional Chinese Medicine" OR "Naturopath*" OR "Holistic medicine" OR "Essential oils" OR "Energy healing" OR "Detoxification" OR "Dietary modification*" OR "Alternative healthcare" OR "Meditation" OR "Natural healing" OR "Ayurveda" OR "Botanical medicine" OR "Wellness coaching") NEAR/15 ("vaccine alternatives" OR "alternative* to vaccine*" OR "vaccine risks" OR "vaccine efficacy" OR "vaccine hesitancy" OR "vaccine refusal" OR "vaccine safety concerns" OR "vaccine misinformation" OR “no forced vaccines”)
10. Vaccine Brands: ("Pfizer" OR "Moderna" OR "Johnson & Johnson" OR "J&J" OR "NovaVax") AND ("vaccine" OR "mRNA" OR "propaganda" OR "hoax" OR “religious exemption” OR “no forced vaccines” OR “unvaccinated” OR “no vax” OR “vaccine mandate” OR "long term effects of vaccine” OR “long term effects of COVID” OR "Long COVID" OR "mask mandate" OR "whistleblowers*" OR "health freedom" OR "free thinker" OR "vaccine safety" OR "vaccine side effects" OR "vaccine risks" OR "vaccine benefits" OR "vaccine efficacy" OR "vaccine effectiveness" OR "vaccine mandates" OR "vaccine hesitancy" OR "vaccine refusal" OR "vaccine confidence" OR "vaccine trust" OR "vaccine safety concerns" OR “vaccine rights” OR “no to vaccines” OR “vaccine mandate” OR "anti-vaxxer” OR “vax” OR “vaxxed” OR “vaccine” OR "vaccine refusal" OR "vaccine misinformation" OR "vaccine skepticism" OR “covid hoax” OR “mask mandate” OR “COVID propaganda”)
11. Vaccine Hesitancy: General U.S.: ("vaccine safety" OR "vaccine side effects" OR "vaccine risks" OR "vaccine benefits" OR "vaccine efficacy" OR "vaccine effectiveness" OR "vaccine mandates" OR "vaccine hesitancy" OR "vaccine refusal" OR "vaccine confidence" OR "vaccine trust" OR "vaccine safety concerns" OR “vaccine rights” OR “no to vaccines” OR “vaccine mandate” OR "anti-vaxxer” OR “vax” OR “vaxxed” OR "vaccine refusal" OR "vaccine misinformation" OR "vaccine skepticism" OR “covid hoax” OR “mask mandate” OR “COVID propaganda” OR “no forced vaccines” OR “unvaccinated” OR “no vax” OR "anti vax" OR "#vaccinesafety" OR "#vaccinesideeffects" OR "#vaccinerisks" OR "#vaccinebenefits" OR "#vaccineefficacy" OR "#vaccineeffectiveness" OR "#vaccinemandates" OR "#vaccinehesitancy" OR "#vaxhesitancy" OR "#vaccinerefusal" OR "#vaccineconfidence" OR "#vaccinetrust" OR "#vaccinesafetyconcerns" OR “#vaccinerights” OR “#notovaccines” OR “#vaccinemandate” OR "#antivaxxer” OR “#vax” OR “#vaxxed” OR "#vaccinerefusal" OR "#vaccinemisinformation" OR "#vaccineskepticism" OR “#covidhoax” OR “#maskmandate” OR “#covidpropaganda” OR “#noforcedvaccines” OR “#unvaccinated” OR “#novax” OR "#antivax" OR "#vaccineconcerns" OR "#vaccinedoubts" OR "#informedchoice" OR "#healthdecisions" OR "#vaccineeducation" OR "#publichealthdebate" OR "#informedconsent" OR "#healthfreedom" OR "#medicalchoice" OR "#vaccineawareness" OR "#vaccinedebate" OR "#vaccineinformation" OR "#healthdecisionmaking" OR "#vaccinediscussion" OR "#vaccineviews" OR "#hesitantbutcurious")
12. Vaccine Hestitancy: Louisiana: ("vaccine safety" OR "vaccine side effects" OR "vaccine risks" OR "vaccine benefits" OR "vaccine efficacy" OR "vaccine effectiveness" OR "vaccine mandates" OR "vaccine hesitancy" OR "vaccine refusal" OR "vaccine confidence" OR "vaccine trust" OR "vaccine safety concerns" OR “vaccine rights” OR “no to vaccines” OR “vaccine mandate” OR "anti-vaxxer” OR “vax” OR “vaxxed” OR “anti vax” OR "vaccine refusal" OR "vaccine misinformation" OR "vaccine skepticism" OR “covid hoax” OR “mask mandate” OR “COVID propaganda” OR “no forced vaccines” OR “unvaccinated” OR “no vax” OR "vaccinated" OR "vaccine" OR sourceName:/r/Coronavirus)

Custom Category on all searches: Exclude: ("RT" OR "QT" OR "Jarred Alwan" OR "car seats" OR "car crashes" OR "beverages industry" OR "nicotine" OR "probiotic toothpaste" OR "indoor car cover" OR "vaping" OR "tobacco" OR "raw milk" OR "alien*" OR "ovarian" OR "El Nino" OR "William Frantz Elementary" OR "Sports arena" OR "indoor car cover" OR "Pasteurised milk" OR "egg freezing" OR "Australia" OR "fake meat" OR "slaughter-free meat" OR "pancytovir" OR "oseltamivir" OR "rat terrier" OR "vaccinated dogs" OR "vaccinated my dogs" OR "TNX-801" OR "EBV-001" OR "epstein" OR "shingles" OR "canine" OR "melanoma" OR "dog" OR "brucellosis" OR "BALB" OR "insulin" OR "march of dimes" OR "snake")
