# Supplementary material for: Understanding Vaccine Hesitancy in Louisiana Through Social Media Listening and Community Feedback: Cross-Sectional Study
Source: JMIR Infodemiology. 2026 May 6;6:e76827. doi: 10.2196/76827 (PMC13148589; doi:10.2196/76827)

Social Media Campaign examples

Figure S1

Social Media Campaign Post 1 – April 22, 2024


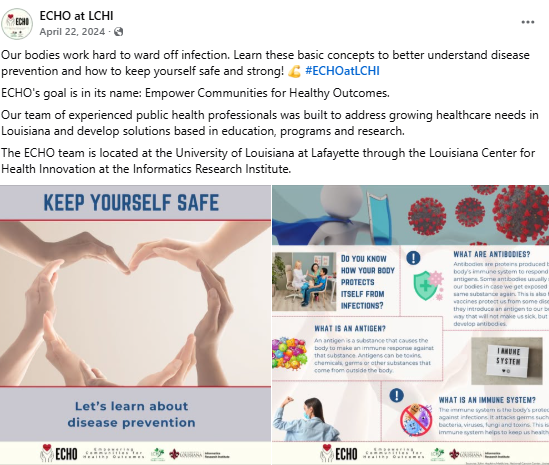


Figure S2

Social Media Campaign Post 11 – May 22, 2024


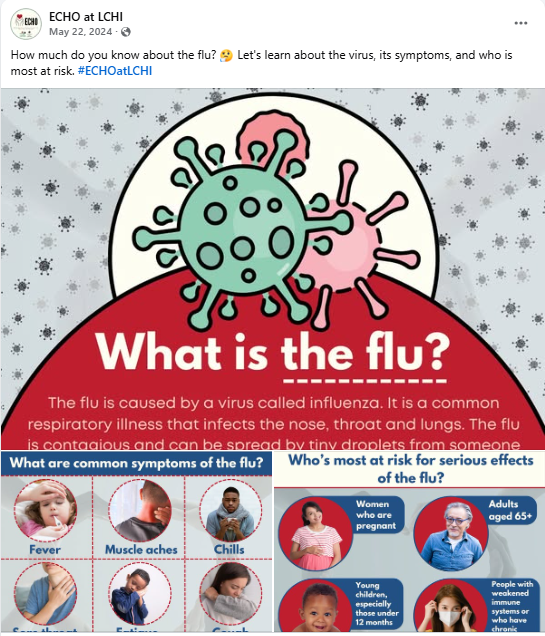


Figure S3

Social Media Campaign Post 13 – May 29, 2024


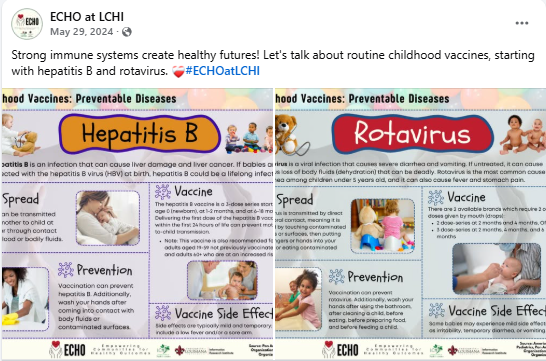


Figure S4

Social Media Campaign Post 13 – June 18, 2024


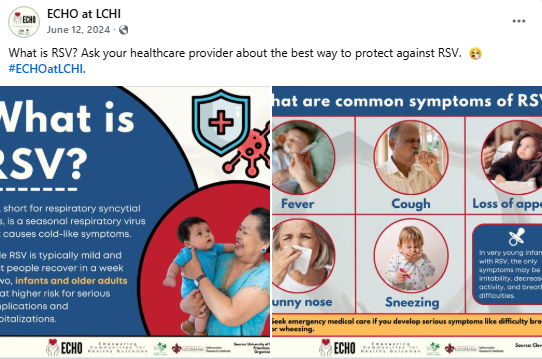

Supplement: Multimedia Appendix 2 [file infodemiology-v6-e76827-s002.docx]
